# Growth responses and genetic variation among highly ecologically diverse spring wheat genotypes grown under seawater stress

**DOI:** 10.3389/fpls.2022.996538

**Published:** 2022-10-14

**Authors:** Ahmed Amro, Shrouk Harb, Khaled A. Farghaly, Mahmoud M. F. Ali, Aml G. Mohammed, Amira M. I. Mourad, Mohamed Afifi, Andreas Börner, Ahmed Sallam

**Affiliations:** ^1^Department of Botany and Microbiology, Faculty of Science, Assiut University, Assiut, Egypt; ^2^Department of Genetics, Faculty of Agriculture, Assiut University, Assiut, Egypt; ^3^Department of Soil and Water Resources, Faculty of Agriculture, Assiut University, Assiut, Egypt; ^4^Resources Genetics and Reproduction, Department Genebank, Leibniz Institute of Plant Genetics and Crop Plant Research (IPK), Gatersleben, Germany; ^5^Department of Agronomy, Faculty of Agriculture, Assiut University, Assiut, Egypt; ^6^Ultrasonic Laboratory, National Institute of Standards, Giza, Egypt

**Keywords:** *Triticum aestivum L*., germination traits, salinity stress, breeding, genetic diversity

## Abstract

Most of the freshwaters worldwide are used for agriculture. Freshwater sources are expected to decline and will not suffice to support the food production needed for the growing population. Therefore, growing crops with seawater might constitute a solution. However, very little work has been done on the effect of seawater stress on wheat, an important cereal crop. The present study aimed to determine whether particular wheat genotypes provided better resistance to seawater stress. A set of 80 highly diverse spring wheat genotypes collected from different countries in Europe, Asia, Africa, North and South America was exposed to 50% seawater stress at the early growth stage. Four seeding shoot and root traits were scored for all genotypes. High genetic variations were found among all genotypes for the epicotyl length (EL), hypocotyl length (HL), number of radicles (NOR), and fresh weight (FW). Eight genotypes with high-performance scores of seedling traits were selected. The correlation analyses revealed highly significant correlations among all traits scored in this study. The strongest correlation was found between the NOR and the other seeding traits. Thus, the NOR might be an important adaptive trait for seawater tolerance. The genetic diversity among all genotypes was investigated based on genetic distance. A wide range of genetic distances among all genotypes was found. There was also a great genetic distance among the eight selected genotypes. In particular, the genetic distance between ATRI 5310 (France) and the other seven genotypes was the greatest. Such high genetic diversity might be utilized to select highly divergent genotypes for crossing in a future breeding program. The present study provides very useful information on the presence of different genetic resources in wheat for seawater tolerance.

## Introduction

Soil salinization is a global and dynamic problem that may increase in the future because of climate change scenarios, e.g., rise in temperature, rise in sea level and impact on coastal areas, and increase in evaporation (Kumar and Sharma, 2020). The predicted increase of the sea level due to the thermal expansion of seawater ranges from 31 to more than 100 cm by the year 2100 (Mimura, 2013). This will reduce the land areas and consequently increase the potential yield losses resulting from the soil salinity. Salinity condition in arid and semi-arid regions occurs due to scanty precipitation and high evaporation (Dehnavi et al., 2020). The deficit in the freshwater supply is compensated by pumping excess ground water, especially in coastal areas (Halder et al., 2022). This situation results in high soluble salt contents (saline soils) and/or high sodium ion (Na^+^) levels (sodic or saline-sodic soils) beneath the crop rooting zone (soil horizon; Sadeghi and Rostami, 2017). This leads to stress that reduces the ability of plants (except halophytes and salt-tolerant crops) to take up water from the soil and causes soil degradation. Ultimately, a significant reduction in crop growth and productivity occurs (Food Agriculture Organization of the United Nations, 2022).

Several salinity management techniques have been deployed to improve the growth efficiency of economic crops under salt stress. Urgent solutions allowing the sustainable use of vital crop vegetation despite the harsh environmental situation are needed. The development of salinity-adapting crops is a realistic solution. Conducting research to find alternative ways to solve salinity problems is essential to meet current and future food demands. Suitable management practices to control salinity problems must be implemented in irrigated fields, irrigation projects, and geohydrologic systems (Tomaz et al., 2021). Among these practices, soil erosion control measures, rainwater harvesting, integrating appropriate plant species, and efficient irrigation methods are routine practices for obtaining suitable and sustainable results (Singh et al., 2018).

Egypt is a unique part of the Middle East located in an arid zone with large flat planes, salt-affected shores, and salt marshes facing the Red Sea and the Mediterranean coasts. These areas are integral components of the Egyptian coastal and inland ecosystems and can serve as important areas for food production. The main salt marshes in Egypt are located in the Red Sea coastal belt in South Sinai and east of the Eastern Desert (Amro et al., 2021). Consequently, the utilization of seawater in Egypt has been the latest endeavor to obtain satisfying agricultural yields and horticultural crops (Rady et al., 2016).

Crop species of Gramineae (including wheat and their cultivars) often differ in their tolerance to salinity. These differences can be assessed through germination percentage and seedling growth in saline conditions. This information is crucial for identifying a suitable salt-tolerant wheat cultivar for cultivation under salt stress conditions (Thabet et al., 2021b). Many studies investigated the response of wheat (*Triticum aestivum* L.) cultivars to salt stress at germination and early seedling-growth stages (Mujeeb-ur-Rahman et al., 2008). Wheat receives a lot of attention because it constitutes an important staple food for at least 36% of the world's population. Indeed, it provides 55% of the carbohydrates, 20% of the calories (Nahar et al., 2017; Seleiman et al., 2021), and essential micro- and macro-nutrients of the human diet. Considering that the international population is predicted to increase by 25% (to reach 10 billion) by 2050 (Halder et al., 2022), the current world production of wheat should be doubled (Food Agriculture Organization of the United Nations, 2022). Achieving this goal is particularly challenged by the greater frequency and number of climate change stressors including salinity.

The germination responses and emerging ability of seeds in a saline environment depend not only on the salt concentration but also on other various biological and genetic factors. Özyazici and Açikbaş (2021) stated that some plants are sensitive to salinity at the early seedling-growth stage because the mechanism of salinity tolerance is not fully developed yet. Salt stress also affects many biochemical characteristics such as antioxidant enzyme activity, proline, protein, and both K+ and Na+ contents in leaves (Ghanaatiyan and Sadeghi, 2015; Sadeghi and Rostami, 2017). The differential suppression of wheat genotypes in salinity conditions might originate from differences in metabolic efficiencies against stress-induced carbon deficit and activities of anti-oxidative enzymes as these have been positively correlated with stress tolerance (Srivastava et al., 2010). Differences in cell membrane stability and macromolecule stability induced by salinity might also be a cause for the different responses (Sadeghi and Robati, 2015).

Screening large germplasms of genotypes from different countries is very useful to identify the ones allowing salt tolerance. Once identified, these genotypes might be used as candidate parents in future breeding programs to produce high-yielding wheat cultivars with high tolerance to abiotic stress. Moreover, the analysis of genetic diversity based on DNA molecular markers might allow a precise selection of truly promising tolerant genotypes to accelerate the breeding programs.

The present study aimed to investigate the impact of genetic variations in highly diverse wheat genotypes collected from different countries on seawater tolerance and to select the one(s) performing the best under seawater stress for phenotypic selection and genetic diversity analysis.

## Materials and methods

### Seawater properties

The pH was measured in a 50% seawater sample using an electric pH meter (Hanna pH 211), and water electric conductivity (EC in dS.m^−1^) was determined using a conductivity meter (4310 JEN WAY). Na^+^ and potassium ions (K^+^) contents were determined with a flame-photometer (Carl-Zeiss DR-LANGE M7D). Methods described by Jackson (1973) were used to determine the concentration of calcium (Ca^+2^), magnesium (Mg^2+^), chloride (Cl^−^), and soluble sulfates (SO${}_{4}^{2-}$) ions. Ca^+2^ and Mg^2+^ contents were volumetrically determined by the titration method against 0.01 N EDTA, and Cl^−^ contents were volumetrically determined against AgNO_3_. The soluble SO${}_{4}^{2-}$ content was estimated by the turbidity method against BaCl_3_ according to the method published by Tabatabai and Bremner (1970).

### Plant material

A set of 48 highly diverse spring wheat genotypes were randomly selected from the population and tested in 10, 40, or 50% seawater stress conditions. This preliminary experiment showed high genetic variation in hypocotyl and epicotyl traits in 50% of seawater. Therefore, the whole population (80 genotypes) was tested in 50% seawater. The genotypes used in this study were highly diverse wheat genotypes collected from different parts of Europe (Supplementary Table 1).

### Experimental layout and trait scoring

In all experiments, 20 seeds/genotype were sown in Petri dishes in three replicates using a randomized complete block design. All experiments were conducted under controlled conditions. The seeds were sterilized using 0.5% Na-hypochlorite (for 2 min) and washed in sterilized water. Then, all genotypes were sown in different seawater concentrations. Finally, all Petri dishes were incubated in dark under normal laboratory conditions (21–23°C and 65–70% humidity) in a Heraeus incubator (Germany) for 10 days. On the 10th day, the epicotyl length (EL, cm), hypocotyl length (HL, cm), and number of radicles (NOR) were measured in at least 10 seeds/genotype. The epicotyl/hypocotyl ratio (EHR) was calculated as the ratio of EL to HL. The seedling's fresh weight (FW, mg) was determined by weighing the shoots of the germinated seeds.

### Statistical analysis

Data were analyzed using the SPSS package (v. 25). The differences in the genotype responses according to their spatial affinities (geographical distribution) and behavior (performance against salinity) were determined. The Shapiro–Wilk test of normality was employed to choose the proper comparison test, and the differences between means were considered significant at *p* < 0.05. Non-parametric tests, i.e., Mann–Whitney *U*-test for comparing two groups and Kruskal–Wallis *H*-test for comparing more than two groups, were used. Correlation analyses of grain germination parameters were carried out. Factorial ANOVA was performed to assess the effect of replication, genotypes, and seawater concentrations on seedling attributes and the effect of their interaction.

ANOVA of all phenotypic data and correlation analyses were carried out to estimate the variance and covariance using PLABSTAT software (Utz, 2011). All graphical presentations of the phenotypic data were performed using R software (R Core Team, 2014).

### Analysis of the genetic diversity

The DNA of the 80 genotypes was sent to Trait Genetics (Gatersleben, Germany) for genotyping-by-sequencing using a 25K Infinium iSelect array. Extensive details on the development of the 25K wheat Infinium array were reported by Aleksandrov et al. (2021). The array genotyping revealed 21,450 single-nucleotide polymorphisms (SNP) markers that were used to calculate the genetic distance among the selected genotypes using the R-package “ade4” as described by Dray and Dufour (2007). The genetic distance was calculated using a simple matching coefficient.

## Results

### Chemical properties of seawater

The analysis of the chemical composition of the 50% seawater sample revealed a high EC value (26.33 dS.m^−1^) and high contents in Na^+^ (6.8 g.L^−1^) and Cl^−^ (10.73 g.L^−1^). According to FAO classification of irrigation water, these values corresponded to high-salinity seawater (Rhoades et al., 1992). Additionally, adequate content in essential nutrients (e.g., Ca^2+^, K^+^, and SO${}_{4}^{2-}$) were measured.

### Variation in growth monitors

**Figure 1:**
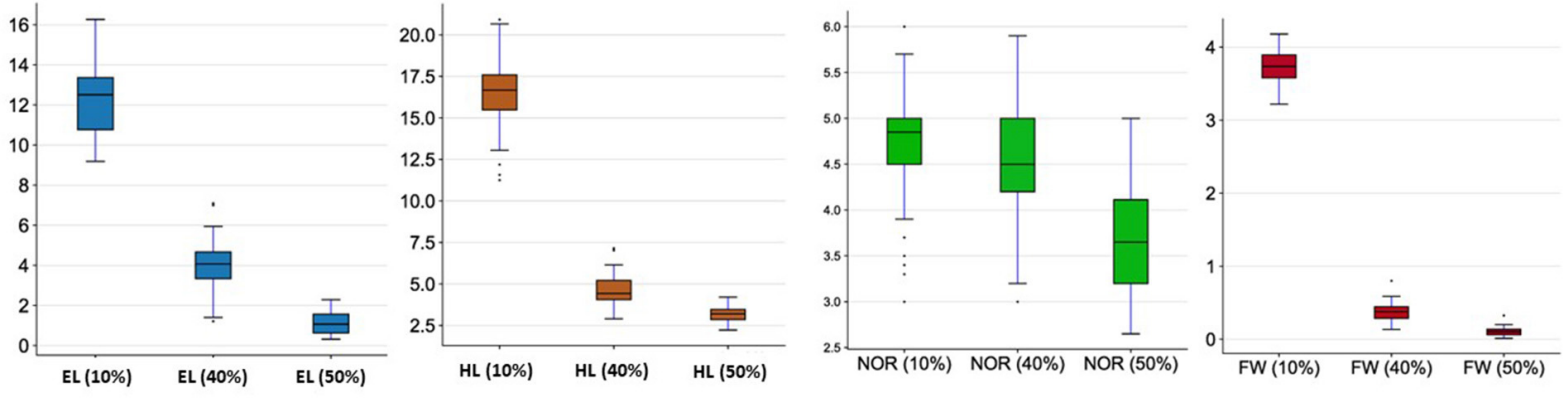
Seedling development under different seawater concentrations (10, 40, and 50%). For abbreviations see Table 1.

The analysis of variation in the selected traits (EL, HL, NOR EHR, and FW) for the 48 genotypes is presented in Table 1. Highly significant differences in all traits were found among the three seawater conditions (10, 40, and 50%). Moreover, the ANOVA revealed significant genetic differences among genotypes for all traits. Highly significant differences in all traits were found among the three seawater conditions (10, 40, and 50%; Figure 1 and Supplementary Table 2). All seedling traits decreased proportionally to the increase in seawater concentration. ELs decreased from 12.25 ± 1.63 cm in 10% seawater to 4.04 ± 1.26 cm and 1.11 ± 0.55 cm when exposed to 40 and 50% seawater, respectively. The same trend was observed for the HL as the mean decreased from 16.63 ± 2.16 cm in 10% seawater to 4.66 ± 0.93 cm and 3.16 ± 0.42 cm in 40 and 50% seawater, respectively. The mean seedling FWs similarly decreased from 3.73 ± 0.23 g in 10% seawater to 0.37 ± 0.13 g in 40% seawater and 0.11 ± 0.05 g in 50% seawater. Interestingly, the NOR was not as much reduced by the different seawater treatments as the other traits were. Indeed, the average NOR was 4.61 in 10% seawater, whereas it was 4.51 and 3.70 in 40 and 50% seawater, respectively.

**Table 1:** The average value of the concentration of major ions contents, EC, and pH in dilute in 50% seawater.

**Property**	**Average**
**pH**	7.12
**EC (dS.m** ^**−1**^ **)**	30.5
**Cation (g.L** ^**−1**^ **)**
Na^+^	6.80
K^+^	0.26
Ca^+2^	1.38
Mg^+2^	0.57
**Anion (g.L** ^**−1**^ **)**
Cl^−^	10.73
SO${}_{4}^{-2}$	1.07

**Figure 2:**
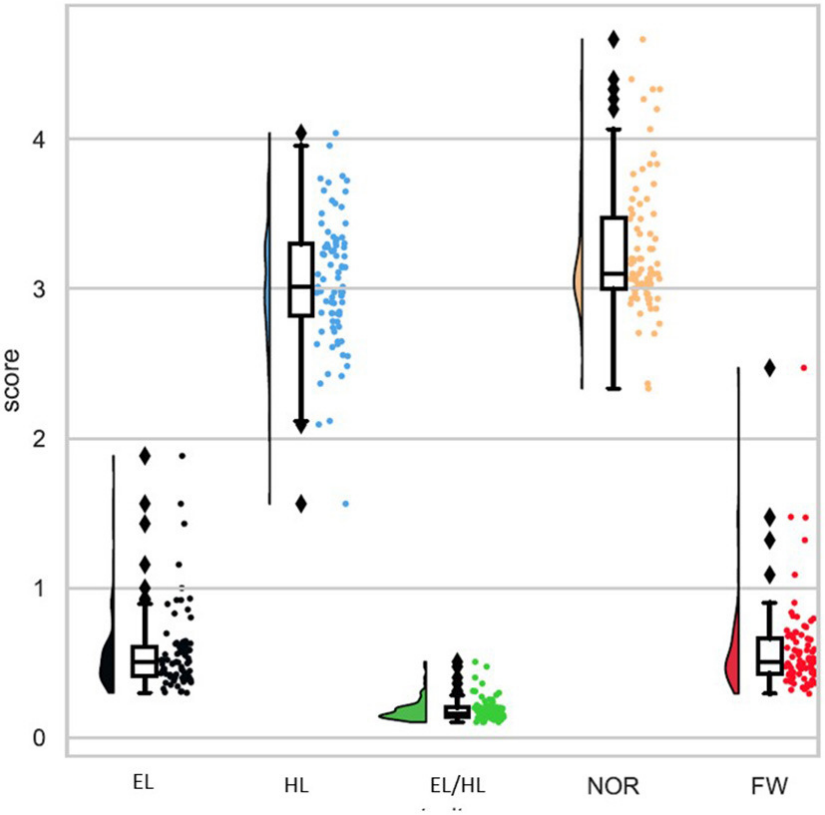
Distribution and variation among all genotypes under 50% seawater. For abbreviations see Table 1.

The variations among the 80 genotypes were thoroughly investigated for 50% seawater. The results of the ANOVA of all previously mentioned traits for 80 genotypes exposed to 50% seawater are presented in Table 2 and supplementary Table 3. Highly significant differences were observed according to replication (R), except for FW. Additionally, highly significant differences were observed among genotypes (G) for all assessed traits. The broad-sense heritability ranged from 0.40 (FW) to 0.84 (HL). The difference in each trait after treatment with 50% seawater due to the genotypic variation is presented in Figure 2. The genotypes performing the best differed according to the trait. For example, ATRI 5692 (Iran) had the greatest EL (1.88 cm). The greatest HL was obtained for ATRI 4563 (Italy). ATRI 10340 (China) had the greatest NOR (4.66) and ATRI 4940 (USA) the greatest FW with 0.24 g.

**Table 2:** Mean square (M.S.) of Epicotyl length (EL), Hypocotyl length (HL), number of roots (NOR), fresh weight (FW), and shoot length/root length ratio under three salt treatments with different concentrations (10, 40, and 50%) in a set of 48 wheat genotypes.

**Source of variance**	**EL**		**HL**		**NOR**		**FW**		**EL/HL**	
	**d.f**.	**M.S**.	**d.f**.	**M.S**.	**d.f**.	**M.S**.	**d.f**.	**M.S**.	**d.f**.	**M.S**.
Treatments (T)	2	1,598.40**	2	2,618.26**	2	12.93**	2	196.20**	2	3.53**
Genotypes (G)	47	2.26**	47	2.96**	47	0.69**	47	0.03**	47	0.07**
TxG	92	1.14	92	1.34	92	0.27	92	0.023	92	0.02
Total	141	–	141	–	141	–	141	–	141	–

^*^P < 0.05.

^**^P < 0.01.

**Figure 3:**
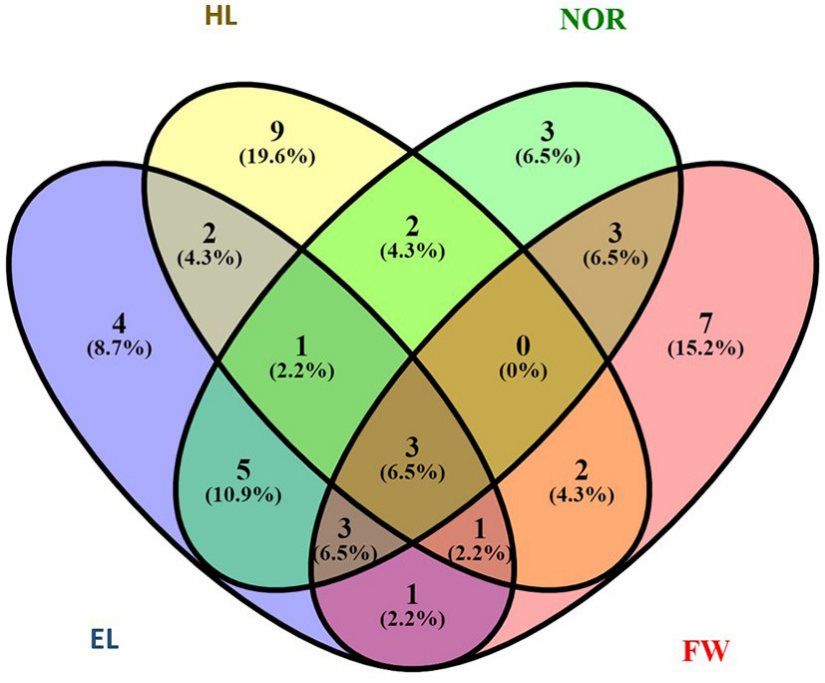
Number of genotypes with high performance.

To determine the best performing genotypes, the 20 genotypes bearing traits with higher values were chosen. Then, the genotypes figuring among these 20 for at least three traits were selected (Figure 3 and Table 3). These criteria were fulfilled by eight genotypes from seven countries: Nepal (2), India (1), Iran (1), France (1), Sweden (1), the USA (1), and Argentina (1). A set of three-common genotypes ATRI 5310 (France), ATRI 5325 (Argentina), and ATRI 2679 (India) were identified as the best performing genotypes for all assessed traits when exposed to 50% seawater.

**Table 3:** Mean square (M.S.) of epicotyl length (EL), hypocotyl length (HL), number of radicles (NOR), and fresh weight (FW) under 50% salt treatment in a set of 80 wheat genotypes.

**Source of variance**	**EL**		**HL**		**NOR**		**FW**		**EL/HL**	
	**d.f**.	**M.S**.	**d.f**.	**M.S**.	**d.f**.	**M.S**.	**d.f**.	**M.S**.	**d.f**.	**M.S**.
Replication (R)	2	0.26**	2	5.13**	2	1.45**	2	8.34	2	0.04**
Genotypes (G)	79	0.11**	79	0.54**	79	0.60**	79	2.03**	79	1.37**
RxG	156	0.02	158	0.09	158	0.09	157	3.84	158	0.92
Total	237	–	239	–	239	–	238	–	239	–
Heritability	0.82		0.84		0.85		0.52		0.66	

^*^P < 0.05.

^**^P < 0.01.

### Variation in seawater tolerance among continents

**Figure 4:**
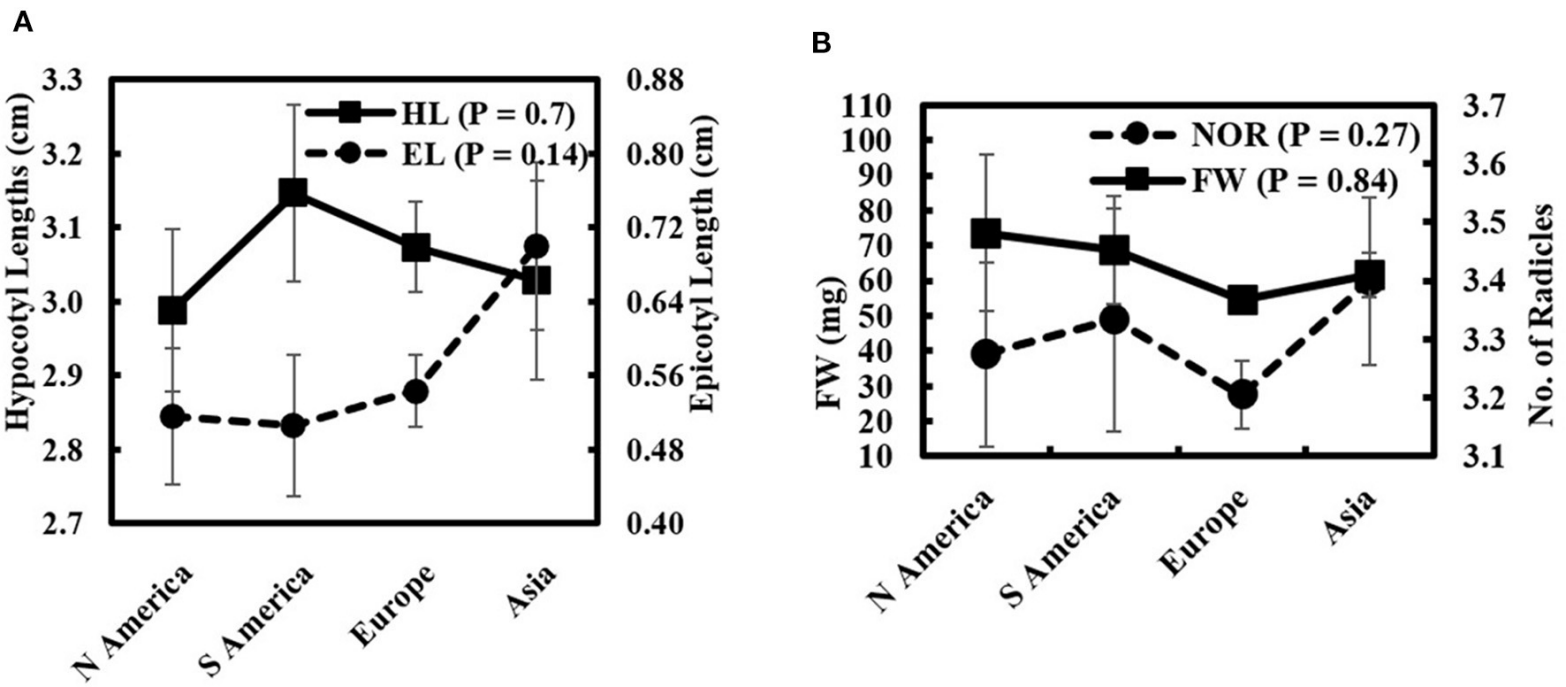
Wheat genotypes attributes means classified according to their global affinities for **(A)** EL and HL and **(B)** FW and NOR under salinity stress (50% seawater).

The comparison of seedling-growth attributes in response to 50% seawater according to their origin is represented in Figures 4A,B. Kruskal–Wallis *H*-test of non-parametric data (normal distribution not assumed) showed no significant differences among continents. The greatest mean HL (3.15 ± 0.12 cm) was measured in South American genotypes. The seedling FW increased in North American genotypes (73.50 ± 22.28 mg). The greatest mean EL and NOR (0.70 ± 0.09 cm and 3.40 ± 0.14, respectively) were recorded in seedlings with Asian genotypes. In contrast, a decrease in the seedling FW and NOR was found in European genotypes exposed to 50% seawater.

### Phenotypic correlation

**Figure 5:**
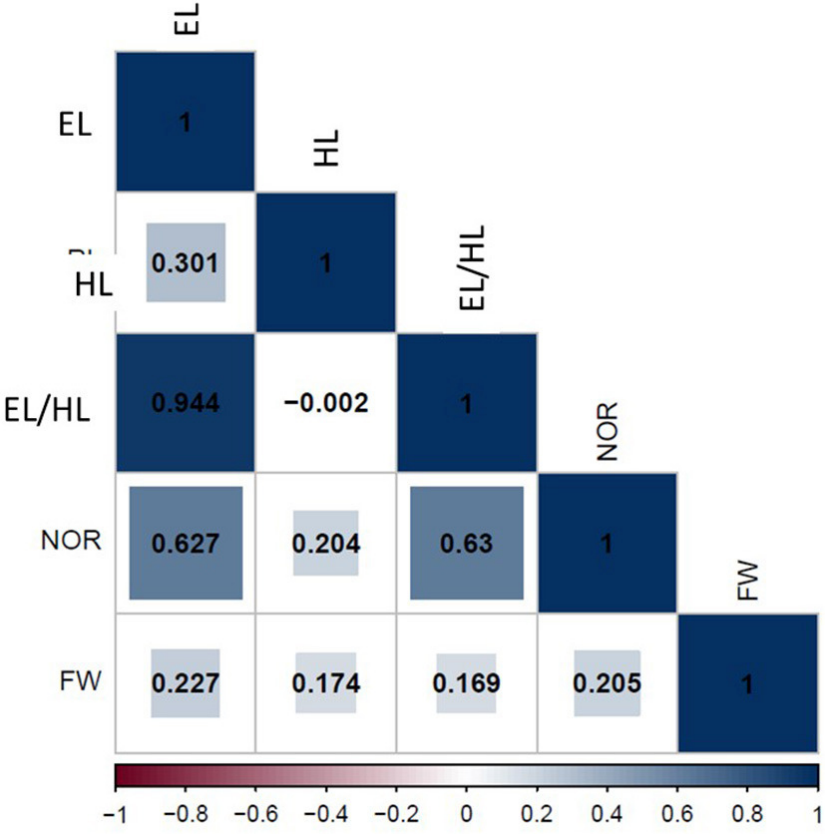
Phenotypic correlation among all traits scored under 50% seawater stress.

The correlation analysis of traits after exposure to 50% seawater is presented in Figure 5. Significant positive correlations were found between EL and EHR (*r* = 0.94^**^), EL and NOR (*r* = 0.63^**^), and EL and FW (*r* = 0.28^*^). There also was a significant positive correlation between HL and NOR (*r* = 0.62^**^) and HL and FW (*r* = 0.17^*^). Additionally, EHR was positively and significantly associated with NOR (*r* = 0.63^**^) and FW (*r* = 0.17^*^). NOR and FW were positively correlated (*r* = 0.21^*^). The strongest positive correlations were found between EL and EHR, EL and NOR, and EHR and NOR.

### Genetic distances among genotypes

**Figure 6:**
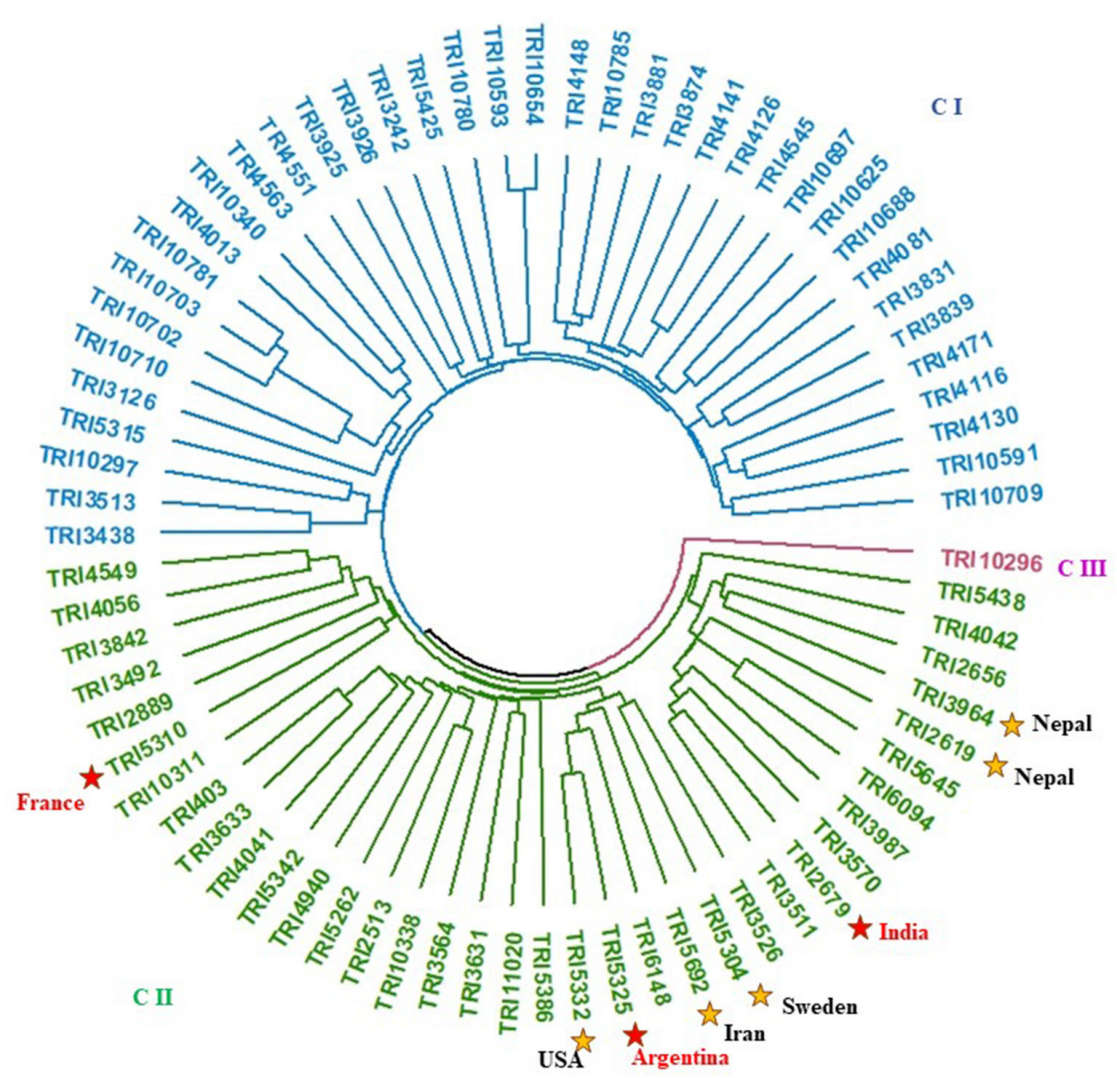
Dendrogram analysis based on genetic diversity among all genotypes.

The genetic distance among the 80 genotypes was calculated to assess the level of genetic diversity in the population (Figure 6 and Supplementary Table 4). The dendrogram analysis revealed two main groups and one genotype, TRI10296 (Mexico). The genetic distance ranged from 0.0988 between TRI10654 and TRI10593 from Cyprus to 0.6696 between TRI3831 (Portugal) and TRI3631 (Canada).

When analyzing the best performing genotypes in a 50% seawater condition, eight genotypes (Table 3) were found in group II (G II.). Notably, TRI5310 (France) was highly distanced from the other selected genotypes. Generally, a high genetic distance was found among the eight selected genotypes and ranged from 0.354 between TRI5332 (USA) and TRI5325 (Argentina) to 00.62998 between TRI5310 (France) to TRI2679 (India).

## Discussion

### Genetic variation and seawater tolerance

Salt-affected soils are found in Australia, China, the former USSR, India, Iran, Bangladesh, Pakistan, Egypt, Iraq, Syria, Turkey Mexico, and the USA (Sanower Hossain and Sultan Ahmad Shah, 2019). More than 833 million hectares of subsoil (30–100 cm) are affected by salinity (Food Agriculture Organization of the United Nations, 2022).

Salinity stress induces a great extent of variations with respect to seed germination and physiological, anatomical, morphological, biochemical, molecular, and genetic impacts. Hence, salinity is one of the factors significantly decreasing wheat production per hectare (Hasseb et al., 2022). Additionally, early stages as germination and seedling growth are critical because they affect all the following stages including grain yield (Mourad et al., 2019; Sallam et al., 2019a; Moursi et al., 2020; Ahmed et al., 2021; Thabet et al., 2021a). It was reported that poor germination and weak seedling growth are major problems that lead to significant deterioration in yield (Sadeghi and Robati, 2015). Moreover, the use of seedlings is relatively simple and cost-effective (Ahmed et al., 2021).

Yildirim et al. (2004) showed that irrigation with seawater with an EC = 15 or 30 dS.m^−1^ significantly decreases the seedling gas exchange and accelerates the respiratory carbon loss, which is coupled with rising in CO_2_ compensation point and a reduction of the photosynthetic assimilation process. The present results revealed that the different seawater treatments had a great impact on all shoot traits scored for all 48 genotypes. Ragab and Taha (2016) reported that increasing salt concentrations decrease seedling shoot dry weight, shoot length, root dry weight, root length, and emergence index in nine Egyptian wheat cultivars. We observed an important and clear variation among seedling traits when using 50% seawater. Therefore, this seawater concentration was selected to test all genotypes of the population.

The high genetic variation among genotypes (*n* = 80) in all traits investigated in this study after exposure to 50% seawater provides great insight for plant breeders and agronomists to select highly performing genotypes in seawater stress conditions. The high broad-sense heritability for HL, EL, and NOR might allow improving these traits for better seawater tolerance. The heritability of FW was lower than that of the other traits. This high genetic variation that existed among genotypes was due to their being diverse and having originated from 33 countries spread on four continents. Screening highly diverse germplasms from semi-arid and arid regions, especially with salt-affected soils, has been highly recommended for selecting promising salt-tolerant genotypes (Sayed, 1985). The diversity in salt tolerance among different species of wheat was greater than that between ploidy levels (Singh and Chatrath, 2022).

As mentioned before, all genotypes in the present investigation were tested under seawater stress conditions. The analysis of 50% seawater indicated that all genotypes were exposed to a high level of Na^+^, K^+^, and Ca^2+^. Most of the previous studies used NaCl to induce salt stress in wheat and other crops. Unfortunately, only a few studies used natural seawater to test the tolerance to salinity of wheat genotypes (Kingsbury and Epstein, 1984; Nassar et al., 2020; Hadia et al., 2022; Kulshreshtha et al., 2022). Two studies assessed the seawater tolerance of wheat genotypes at the germination and seedling stages (Kingsbury and Epstein, 1984; Hadia et al., 2022). Therefore, the present work provides very useful information on the tolerance to seawater stress at early growth stages and might contribute to improving seawater tolerance in wheat. Kingsbury and Epstein (1984), exposed a set of 312 hexaploid wheat varieties to 50% seawater and found 29 salt-resistant lines (9%) with vigorous germination. Here, all the 80 genotypes germinated, and no significant differences were found in the germination percentage after treatment. However, significant variation was observed in shoot and root traits. Out of the 80 genotypes, 8 (10%) showed vigorous growth under 50% seawater condition. Therefore, these eight genotypes might be used for developing seawater-tolerant strains.

Two wheat genotypes were tested for their tolerance to seawater at 0.75% = 13.053 mS.cm^−1^, 1.5% = 24.695 mS.cm^−1^, and 3% = 46.253 mS.cm^−1^ by Hadia et al. (2022) who reported significant differences in coleoptile weight, radicle weight, NOR, coleoptile length, radicle length, radicle length to coleoptile length ratio, and total seedling length. The reduction of the radical number was less important than the reduction in other traits investigated in their study (Hadia et al., 2022). This agrees with the present results as the NOR was not as clearly reduced as the other traits. The more genotypes are investigated for target traits, the higher the genetic variation and, consequently, the better the selection. Therefore, the NOR might be an important trait for enhancing salt tolerance in wheat as it was the least affected by different concentrations of seawater. Shoot growth is more sensitive to salt stress than root growth because the accumulation of Na^+^ and/or Cl^−^ at toxic levels affects the photosynthetic capacity, resulting in less supply of carbohydrates to the young leaves and further reducing the shoot growth rate (Munns and Tester, 2008).

To precisely select the genotypes performing the best when exposed to 50% seawater, all genotypes were sorted from the highest to lowest values for each trait. Eight genotypes were found among the 20 genotypes with the highest values for at least three traits. The selection was based on the performances for multiple traits to identify true tolerant genotypes (Sallam et al., 2015, 2018; Bhavani et al., 2021; Ghazy et al., 2021; Mondal et al., 2021; Mourad et al., 2021; Hasseb et al., 2022). Interestingly, the eight selected genotypes displayed a good seawater tolerance and might be used as a basis for improving salt tolerance in wheat at early growth stages. It is recommended to perform the selection at early growth stages in controlled conditions rather than in field conditions because screening the germplasm at the seedling stage may reduce the number of lines to test at another growth stage (Sayed, 1985; Sallam et al., 2016; Abou-Zeid and Mourad, 2021).

Here, the genotypes originated from different geographical regions. Therefore, it was worth comparing the performances under seawater stress conditions of genotypes from one continent with those of genotypes originating from other continents. There were no significant differences in all traits among groups from different continents. However, Asian genotypes had, on average, the greatest NOR and EL compared to those from the continents. In the study from Sayed (1985) a set of 5,072 wheat germplasm lines at different ploidy levels was exposed at the seedling stage to different salt concentrations with different electrical conductivities of 0.8 (control), 12.5, 18.75, and 25.0 dS.m^−1^. He found 442 genotypes with more than 70% surviving seedlings when tested for whole-life cycle survival. The largest groups of tolerant genotypes were from the USA and Egypt (Sayed, 1985). Furthermore, the widest variability among genotypes was observed in seedlings originating from the USA, Mexico, and Egypt.

### Phenotypic correlations

In the population (*N* = 80 genotypes) tested in the present study, important significant correlations among seedling traits were observed. Root traits were highly associated with shoot traits in the seawater stress condition. Compared with HL, the NOR was significantly more correlated with EL and FW. Therefore, the NOR seemed to be a trait less affected by seawater stress than HL. Breeding to increase the NOR as an adaptive trait might allow to improve seawater tolerance, especially as root traits play an important role in the ability of plants to survive irrigation with seawater (Trimble, 2020). It was reported that salt stress inhibit the growth and number of primary and lateral roots causing a significant decrease in the root zone (Julkowska et al., 2014; Koevoets et al., 2016). Julkowska et al. (2014) found that no effect of salt stress on lateral root density, suggesting that the reduction in the number of lateral roots is related to the inhibition of primary root growth (Julkowska et al., 2014).

The positive correlations among HL, EL, and FW suggested that shoot water gain or loss is a direct consequence of the water absorption capacity of the root systems because of the high osmotic potential coupled with salt stress around the plant rooting zone (Oyiga et al., 2018). This significant positive correlation among traits allowed the selection of genotypes with high seedling traits in 50% seawater conditions.

Hasseb et al. (2022) studied the correlation among shoot and root traits in a set of 138 wheat genotypes at early growth stages exposed to 175 mM NaCl. They found no or weak correlations among shoot and root traits in wheat. Additionally, no or weak correlations between shoot and root traits were reported in barley subjected to NaCl stress (Moursi et al., 2020). Thus, the correlation among traits may depend on the plant material and the salt concentration.

### Using genetic diversity among genotypes to improve seawater tolerance

Understanding the level of genetic diversity existing among the genotypes of the germplasm is key to genetically improve target traits (Babu et al., 2014; Salem and Sallam, 2016; Eltaher et al., 2018; Sallam et al., 2019b; Mourad et al., 2020). The genetic distance among the 80 genotypes was calculated using 12,390 SNP markers. Different degrees, extending from low to high, of genetic diversity were found among the genotypes. The maximum genetic diversity was achieved when collections of germplasms with genetic variability were from widely different geographic origins. The genetic diversity among the eight wheat genotypes with the greatest traits under seawater stress ranged from 0.354 to 0.692. Although there were two genotypes from Nepal (TRI2619 and TRI2679) among these selected genotypes, the genetic distance among them was 0.508, which was greater than the minimum genetic distance of 0.354 found between TRI5332 (USA) and TRI5325 (Argentina). Thus, crossing between TRI5332 and TRI5325 might not be useful and other candidate genotypes might be selected. Interestingly, TRI5310 from France was highly distanced from all genotypes and including this genotype for crossing in breeding programs might be fruitful. Therefore, it is very important to estimate the genetic distance for a better selection of genotypes to cross. This high genetic diversity can be utilized to produce wheat cultivars with high tolerance to seawater stress, even to higher seawater concentrations, by crossing highly divergent genotypes (Eltaher et al., 2021).

Another important step included in the genetic analysis was to confirm the diversity of the genotypes in the target population. These genotypes were collected from farmers from different countries. As some genotypes were collected from different parts of a given country, they had different accession numbers. Thus, duplicate genotypes with the same genetic makeup could be found. The analysis of genetic diversity can detect such duplicates, and redundant genotypes can be excluded as they will affect the selection procedures for improving target traits.

The analysis of genetic diversity performed here was very useful in selecting true and promising high-performance genotypes with a high level of genetic diversity. Using these genotypes in breeding programs might accelerate and facilitate the achievement of goals such as obtaining strains resistant to seawater. Such selected genotypes (Table 4) with high performance under salt stress can be utilized in Egypt not only to improve seawater tolerance but also to expand the circle of genetic diversity of wheat in Egypt. The 80 genotypes tested in this study have good growing conditions in Egypt (Ahmed Sallam, personal communications).

**Table 4:** List of superior genotypes based on at least three out of the four studied traits, Epicotyl length (EL) and Hypocotyl length (HL), number of roots (NOR), and fresh weight (FW).

**Genotype**	**Country**	**Superior studied trait**			
		**EL**	**HL**	**NOR**	**FW**
ATRI 5310*	France	1.563^+^	3.657^+^	4.067^+^	1.4713^+^
ATRI 5325*	Argentina	0.920^+^	3.310^+^	4.400^+^	0.796^+^
ATRI 2679*	India	0.830^+^	3.343^+^	3.533^+^	0.705^+^
ATRI 2619	Nepal	0.620^+^	3.503^+^	3.767^+^	0.458^−^
ATRI 3964	Nepal	0.647^+^	3.380^+^	3.167^−^	1.320^+^
ATRI 5692	Iran	1.883^+^	3.257^−^	4.267^+^	0.814^+^
ATRI 5332	USA	1.000^+^	3.277^−^	3.500^+^	0.808^+^
ATRI 5304	Sweden	0.803^+^	3.073^−^	4.333^+^	0.717^+^

^*^Genotypes that are superior based on all the four studied seedling traits. ^+^refers that the genotype was among the 15 highest performance genotypes under seawater stress for the respective trait.

In conclusion, analyzing germplasms with high genetic variation and different seawater tolerances is very useful in identifying genotypes that might be used in future breeding programs. Moreover, the evaluation of tolerance to seawater at early stages (i.e., seedling stage) will help to reduce screening efforts in field conditions. Seedling traits including shoots and roots allowed to distinguish between high- and low-performance genotypes in seawater stress conditions. Three genotypes had high-performance scores for all traits investigated in the present study. Incorporating the analysis of genetic diversity contributed to the selection of candidate parents for crossing to produce wheat cultivars having high tolerance to seawater. The present data provide useful information on seawater tolerance at the seedling stage in wheat, particularly as very few studies focused on this research point.

## Data availability statement

The original contributions presented in the study are included in the article/Supplementary materials, further inquiries can be directed to the corresponding author.

## Author contributions

AM designed the study, analyzed data, and wrote the manuscript. SH, MMA, and AGM collected the phenotypic data and helped in data analysis. KY analyzed seawater under different concentrations. AMIM helped in collecting data and analyzing the phenotypic data data. MA helped in data collection and analysis. AB provided the germplasm, discussed the results, and helped in editing the paper. AS designed the study, supervised the experiment, and wrote the manuscript. All authors contributed to the article and approved the submitted version.

## Funding

This paper is based upon work supported by Science, Technology and Innovation Funding Authority (STDF) under grant number 43694. Costs for open access publishing were partially funded by the Deutsche Forschung Gemeinschaft (DFG, German Research Foundation grant 491250510). This work was financially partial supported by Alexander von Humboldt Foundation.

## Conflict of interest

The authors declare that the research was conducted in the absence of any commercial or financial relationships that could be construed as a potential conflict of interest.

## Publisher's note

All claims expressed in this article are solely those of the authors and do not necessarily represent those of their affiliated organizations, or those of the publisher, the editors and the reviewers. Any product that may be evaluated in this article, or claim that may be made by its manufacturer, is not guaranteed or endorsed by the publisher.
