# Erratum: Growth responses and genetic variation among highly ecologically diverse spring wheat genotypes grown under seawater stress

**DOI:** 10.3389/fpls.2022.1089660

**Published:** 2022-11-18

**Authors:** Frontiers Production Office

**Affiliations:** Frontiers Media SA, Lausanne, Switzerland

**Keywords:** *Triticum aestivum L*., germination traits, salinity stress, breeding, genetic diversity

Due to a production error, an author’s name was incorrectly spelled as “Khaled Youssef”. The correct spelling is “Khaled A. Farghaly”. The publisher apologizes for this mistake.

The original version of this article has been updated.

